# Spatial Distribution and Population Estimation of Dogs in Thailand: Implications for Rabies Prevention and Control

**DOI:** 10.3389/fvets.2021.790701

**Published:** 2021-12-21

**Authors:** Weerapong Thanapongtharm, Suwicha Kasemsuwan, Vilaiporn Wongphruksasoong, Khemmapat Boonyo, Tanu Pinyopummintr, Anuwat Wiratsudakul, Marius Gilbert, Kansuda Leelahapongsathon

**Affiliations:** ^1^Department of Livestock Development (DLD), Bangkok, Thailand; ^2^Faculty of Veterinary Medicine, Kasetsart University, Nakhon Pathom, Thailand; ^3^Department of Clinical Sciences and Public Health and the Monitoring and Surveillance Center for Zoonotic Diseases in Wildlife and Exotic Animals, Faculty of Veterinary Science, Mahidol University, Nakhon Pathom, Thailand; ^4^Spatial Epidemiology Lab. (SpELL), University Libre de Bruxelles, Brussels, Belgium; ^5^Fonds National de la Recherche Scientifique (FNRS), Brussels, Belgium

**Keywords:** dog survey, rabies, random forest model, stray dogs, Thailand

## Abstract

Poor management of dog populations causes many problems in different countries, including rabies. To strategically design a dog population management, certain sets of data are required, such as the population size and spatial distribution of dogs. However, these data are rarely available or incomplete. Hence, this study aimed to describe the characteristics of dog populations in Thailand, explore their spatial distribution and relevant factors, and estimate the number of dogs in the whole country. First, four districts were selected as representatives of each region. Each district was partitioned into grids with a 300-m resolution. The selected grids were then surveyed, and the number of dogs and related data were collected. Random forest models with a two-part approach were used to quantify the association between the surveyed dog population and predictor variables. The spatial distribution of dog populations was then predicted. A total of 1,750 grids were surveyed (945 grids with dog presence and 805 grids with dog absence). Among the surveyed dogs, 86.6% (12,027/13,895) were owned. Of these, 51% were classified as independent, followed by confined (25%), semi-independent (21%), and unidentified dogs (3%). Seventy-two percent (1,348/1,868) of the ownerless dogs were feral, and the rest were community dogs. The spatial pattern of the dog populations was highly distributed in big cities such as Bangkok and its suburbs. In owned dogs, it was linked to household demographics, whereas it was related to community factors in ownerless dogs. The number of estimated dogs in the entire country was 12.8 million heads including 11.2 million owned dogs (21.7 heads/km^2^) and 1.6 million ownerless dogs (3.2 heads/km^2^). The methods developed here are extrapolatable to a larger area and use much less budget and manpower compared to the present practices. Our results are helpful for canine rabies prevention and control programs, such as dog population management and control and rabies vaccine allocation.

## Introduction

The poor management of dog populations, especially stray dogs, may result in different problems that exist in many countries. These problems are complexly related to human and animal health, welfare, socio-economics, politics, and religion. According to the World Organization for Animal Health (OIE), a “stray dog” was defined as “any dog not under direct control by a person or not prevented from roaming” (1). The sources of stray dogs may come from the following: (i) owned dogs that roam freely; (ii) abandoned owned dogs, including their puppies born from uncontrolled breeding; and (iii) ownerless dogs that reproduce successfully (1). Stray dogs are associated with the transmission of a number of zoonotic pathogens, dog bite injuries, and road traffic accidents (2). Many of these dogs may be packed and claim a street, which they beg for food, and are a general nuisance to local people and tourists (3). Dog bites are a serious public health problem in many countries. Many studies suggest that dog bites account for tens of millions of injuries annually (4). In Thailand, no <1 million people are bitten by animals annually, and 97% of animal bite injuries are dog bites (5). The majority of dog bites are stray dogs (6). Most bites are delivered by male dogs (7). In addition, bitches with puppies may be aggressive and bite people who approach their litter (8). Other negative impacts caused by stray dogs living in the city environment may include noise pollution, fecal garbage, and traffic accidents. These have been previously observed in different cities in the USA (9). Moreover, previous reports have shown that stray dogs have become a serious public administration problem in many Asian countries, such as China (10), India (11), Bangladesh (12), Indonesia (13), and Cambodia (14). Thailand has been facing stray dog problems for a long time. The rising number of stray dogs has raised concerns regarding human hygiene and public health. Similar problems have also been found, as some pets have been abandoned and become strays (3). In Thailand, dog-keeping practices and duties of responsible ownership vary depending on the cultural setting (15). In urban areas, the majority of owned dogs were frequently confined, but were sometimes allowed to roam. In rural areas, dogs were allowed to roam freely in the village and reproduce with other dogs. People will abandon unwanted dogs and puppy litters in public places, such as temples and universities. Most stray dogs are being fed and looked after by the surrounding community or dog caretakers; however, they have limited capabilities to capture or restrain these dogs (16). Therefore, these dogs cannot be vaccinated or sterilized, and are responsible for sustaining endemic rabies.

One of the most life-threatening issues arising from poor dog population management is dog-mediated rabies. Rabies is a bullet-shaped virus belonging to the genus *Lyssavirus* of the family *Rhabdoviridae* (17). Rabies is a fatal disease once individuals are symptomatic. In Thailand, rabies is endemic and has been listed as an important zoonotic disease (18, 19). Forty-six people died from rabies between 2010 and 2015, and more than 600,000 post-exposure prophylaxis treatments are provided annually (20).

In developed countries, wild animals have been identified as important maintenance hosts for rabies. For example, raccoons, skunks, and bats accounted for 92.4% of animal rabies reported in the USA in 1998 (21). Foxes and raccoons were responsible for 72.1% of animal rabies reported in Europe in 2000 (21). Nonetheless, the scenarios are completely different in developing countries. In Africa and Asia, dog is identified as the main reservoir for rabies (22–24). It was estimated that rabies causes over 59,000 deaths every year in these countries (25) and most of the cases were dog-mediated (26). In Thailand, dog rabies cases have been continuously reported in all regions. From the surveillance system of the Department of Livestock Development between 2013 and 2020, from a total of 58,651 samples, 4,239 tested positive for rabies. The highest number of animal rabies reported was in 2018 (15.3%; 1,476/9,643), and most of the rabies-positive animal samples (87.2%; 1,287/1,476) were retrieved from dogs (27). Thus, dogs are important maintenance hosts of animal rabies in Thailand that continuously spread the virus to humans and other animals. A more comprehensive understanding on the different aspects of dogs living in these areas is crucially needed for a better dog population management. Furthermore, WHO, OIE, FAO and the Global Alliance for Rabies Control have set a global target of “Zero by 30” to end human deaths from dog-mediated rabies by 2030, in line with Thailand's strategic plan under “Saving Animals and Human Lives from Rabies Project” to make Thailand rabies-free through various approaches. A better understanding of the dog population will ultimately help in tailoring strategic plans for rabies prevention and control in a more sustainable direction and encourage the achievement of our 2030 rabies elimination goal.

Nonetheless, a wide variety of data are required, such as the number of dogs and the distribution and ecology of dog populations in a particular area. These data are helpful for dog population management, rabies control, and intervention adaptation (1). For a precise analysis, dog populations should be classified according to ownership status and levels of movement restriction. In general, the number of dogs is derived from different sources, such as household surveys and mark-recapture techniques (28, 29). However, household surveys are labor-intensive, and mark-recapture techniques are not applicable to very large areas (1). Moreover, other factors affecting the living conditions of dogs, including food, shelter, water, and human activities should be considered in dog population estimation and distribution (30–32). Therefore, this study aimed to describe the characteristics of dog populations in Thailand, explore the spatial distribution and relevant ecological factors, and estimate the number of dogs in different population types across the country.

## Materials and Methods

### Study Areas

**Figure 1:**
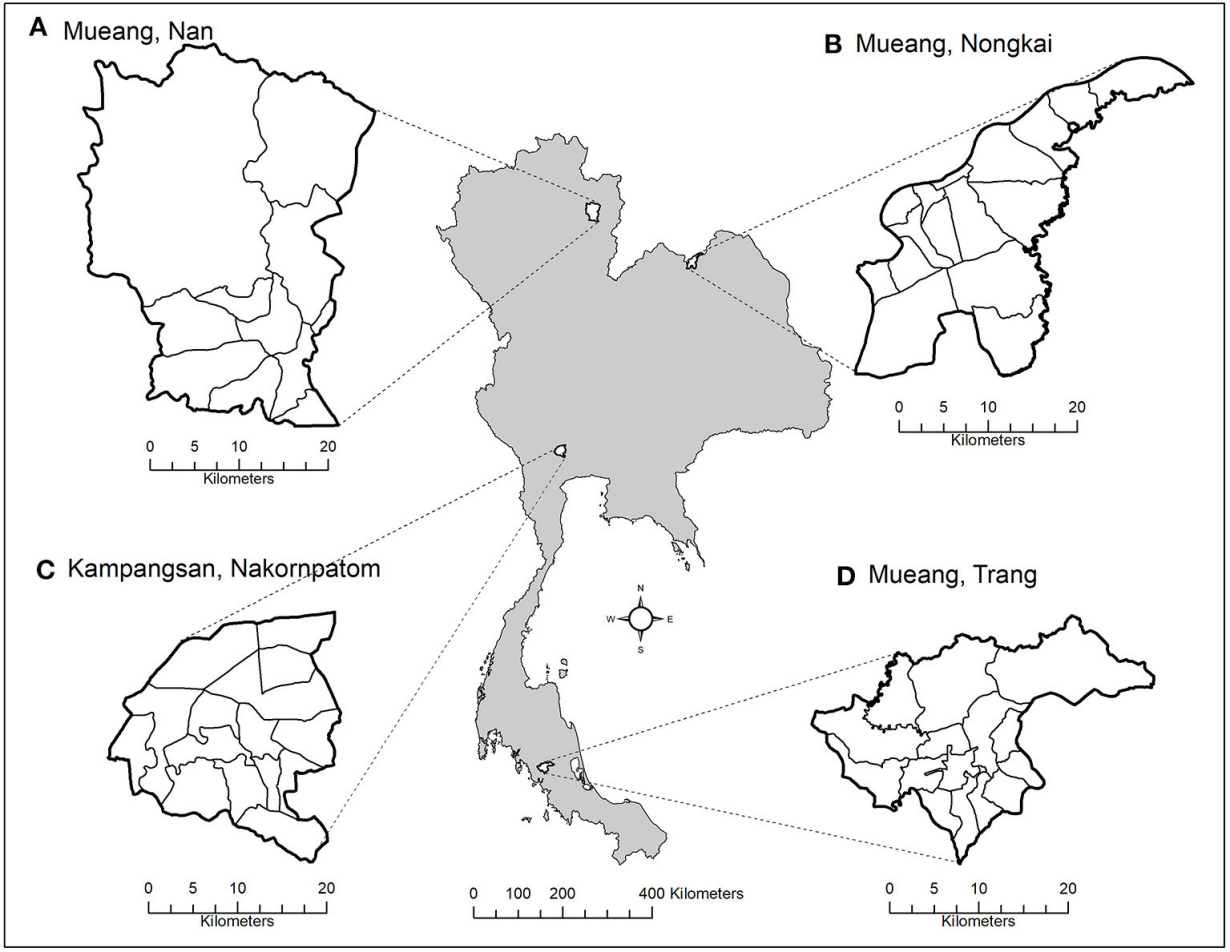
Study areas. The study areas include Mueang Nan district, Nan province **(A)**, Mueang Nong Khai district, Nong Khai province **(B)**, Kampangsan district, Nakhon Pathom province **(C)**, and Mueang Trang district, Trang province **(D)**.

We purposively selected four districts representing the main region of Thailand (Figure 1), including Kampangsan district, Nakhon Pathom province (central region); Mueang Nong Khai district, Nong Khai province (northeastern region); Mueang Nan district, Nan province (northern region); and Mueang Trang district, Trang province (southern region). In each district, the area was divided into grids with 300-m resolution, and at least 10% of the grids (approximately 400 grids) in each district were selected by simple random sampling for dog surveys.

### Dog Surveys

**Figure 2:**
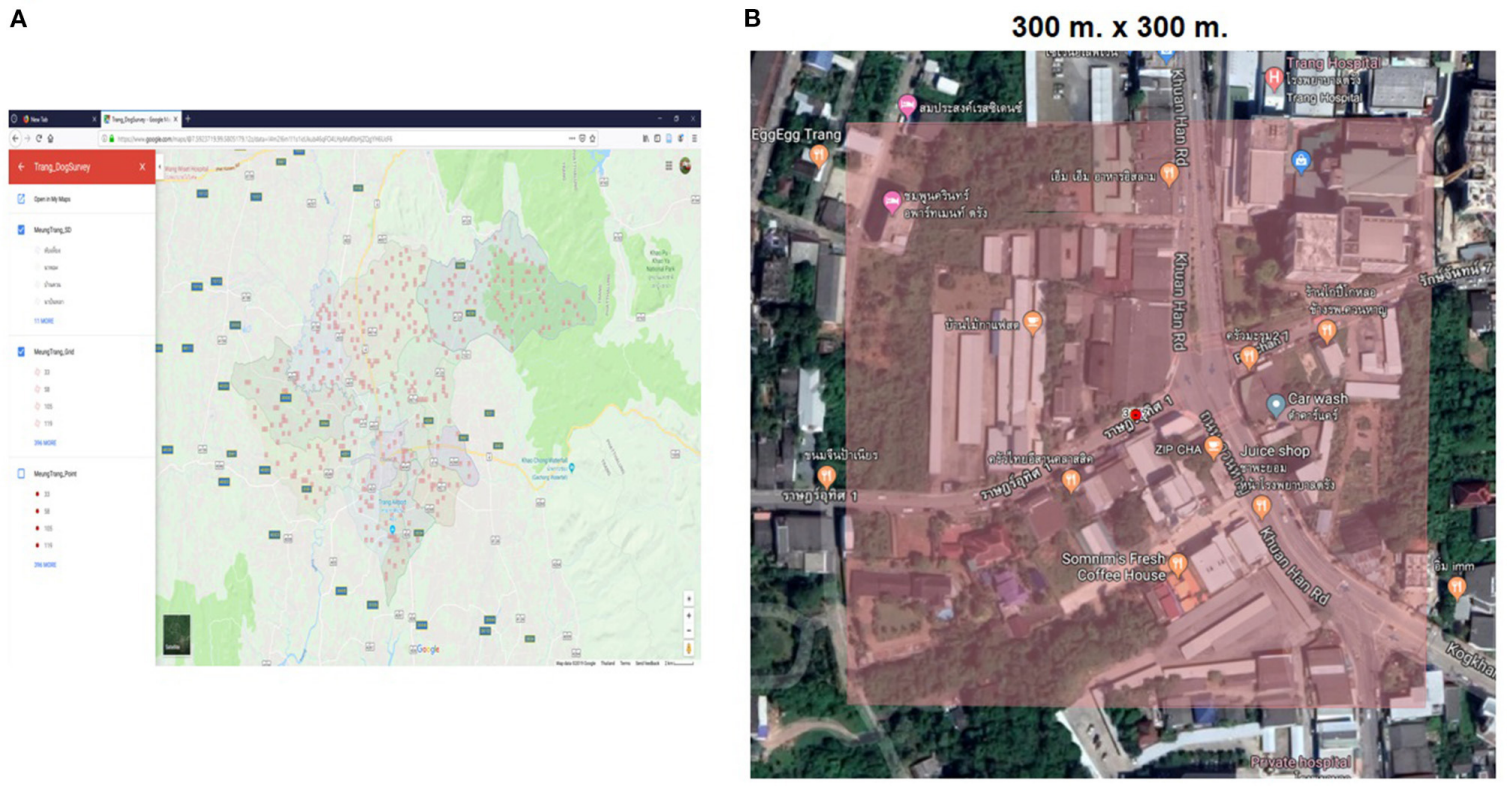
Surveyed grids. Using a Google map to navigate the selected grids **(A)**, which buildings, and areas within a grid (red color) were surveyed **(B)** (33).

The dog survey was conducted between May 2018 and September 2019. In each survey team, a navigator and an observer were recruited. The navigator used Google Map application (Google LLC, California, United States) equipped on their mobile phone to locate the area within the selected grids (Figure 2). Meanwhile, the observer counted the number of dogs and asked owners, neighbors, or witnesses on the ownership status and movement restriction of each dog. Ownership was divided into owned dogs (usually cared for) and ownerless dogs (not cared for or unusually cared for). The movement restriction of owned dogs was characterized as confined (raised only inside the house), independent (lived independently all the time), semi-independent (raised inside and independently), and unidentified (the observer found dogs in the household but no one informed data). The ownerless dogs were characterized into community (fed but not usual) and feral dogs (not fed by anyone).

### Data Analysis

The collected data obtained from the surveys were then used to describe the characteristics of the dog populations in Thailand, including ownership patterns and movement restrictions. Subsequently, these data were used to model the spatial distribution of the dog populations and to predict the number of dogs in each area and the entire country.

We used a random forest (RF) model to quantify the association between the dependent variables (owned and ownerless dogs) and predictor variables. The predictor variables included demographic data (human population), land use (aquaculture, community, residential areas, field crop, and horticulture), and proximity to food sources (Buddhist temples, schools, and roads). A human population density raster map at 100-m resolution was obtained from the WorldPop project (34). Land use data was provided by the Land Development Department (35). We defined “residential areas” as a housing space where people and their companion animals live, whereas “community area” is where public places such as markets, temples, schools, and commercial buildings are located. All predictor layers were transformed into raster maps with a resolution of 300 m. All predictor layers were extracted and divided into five datasets, as listed in Table 1.

**Table 1:** Datasets for the random forest model.

**Model**	**Training site**	**Test site**
I	Northeast, North and South	Central
II	Central, North and South	Northeast
III	Central, Northeast and South	North
IV	Central, Northeast and North	South
V	Central, Northeast, North and South	No test set

We modeled the datasets in two separate parts: count data and binary data (presence/absence). This approach is called “a two-part model” or “a zero-altered model” or “a hurdle model” (36, 37). First, we selected grids with dog presence and modeled them as a quantitative RF (with count data). We then evaluated the predictive power of the models using two statistical metrics, the correlation coefficient (COR), and the root mean square error (RMSE) to quantify the goodness of fit (GOF) between the observed values of the model sets and predicted values. Second, we defined grids with dog presence as 1 and 0 as otherwise. A binary RF is then applied. Two other statistical metrics, the area under the curve (AUC) of the receiver operating characteristic plots, and COR were used to evaluate the predictive power of the binary RF models. Finally, we combined the predictive values of both approaches to produce a final map of predictive values and evaluated them with the test sets using the COR and RMSE. The packages “randomForest” (38) and “hydroGOF” (39) equipped in program R (R Core Team, Vienna, Austria) were employed for the RF model and goodness of fit estimation, respectively. The total numbers of dogs in the whole country were calculated using ArcGIS 10.2 (Esri, California, United States), separated by the five models.

## Results

A total of 1,750 grids were surveyed in this study (945 grids with dog presence and 805 grids with dog absence). Among the observed dogs, 12,027 (86.6%) were owned and 1,868 (13.4%) were ownerless. The ratio between owned and ownerless dogs was 6.4:1. Among the owned dogs, 25% were confined, 51% were independent, 21% were semi-independent dogs, and the rest were unidentified. Among the ownerless dogs, 72% were feral, and the remaining were community dogs. Details of the surveyed dogs are presented in Table 2.

**Table 2:** Descriptive analysis of surveyed dogs.

**Study areas**	Grids (P/A)^*^	**Owned dogs**					**Ownerless dogs**			**Total**
		**Confined**	**Independent**	**Semi**	**Unidentified**	**Total**	**feral**	**community**	**Total**	
Kampangsan district (Nakhon Pathom province)	550 (459/91)	1,572	3,309	1,690	263	6,834	1,052	134	1,186	8,020
Mueang Nong Kai district (Nong Kai province)	400 (206/194)	743	1,467	461	26	2,697	87	42	129	2,826
Mueang Nan district (Nan province)	400 (44/356)	90	213	129	2	434	0	4	4	438
Mueang Trang district (Trang province)	400 (236/164)	553	1,175	264	70	2,062	209	340	549	2,611
Total	1,750 (945/805)	2,958	6,164	2,544	361	12,027	1,348	520	1,868	13,895

* *P stands for the number of grids with dog presence and A stands for the number of grids with dog absence*.

**Figure 3:**
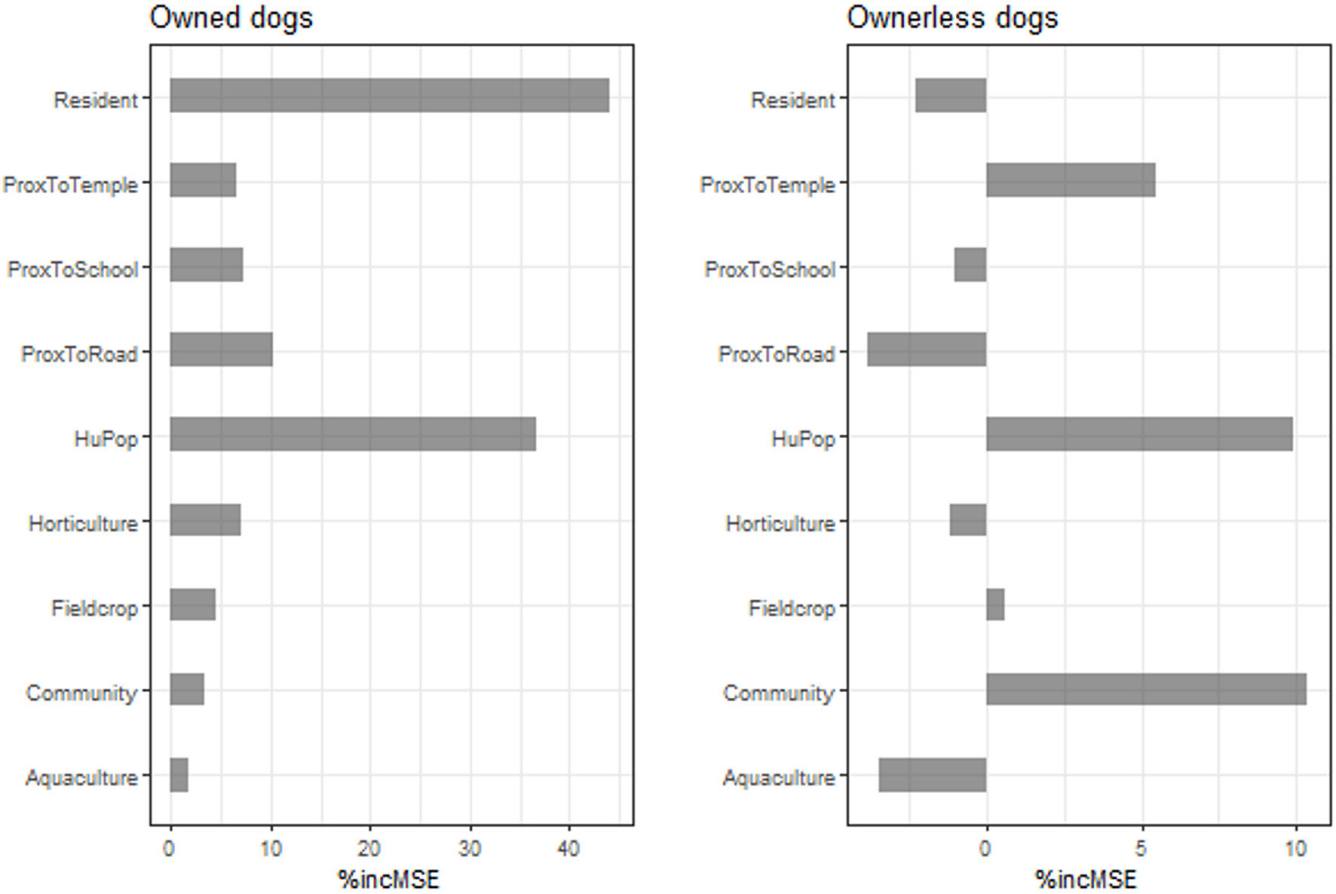
Graphs showed the importance of variable. The percent increase of MSE of the factors influencing the distribution of owned dogs **(left)** and ownerless dogs **(right)**.

The variable importance of the predictors is shown in Figure 3 and Table 3. The three predictors that mostly influenced the distribution of owned dogs in the count models were the resident area, followed by the human population density and proximity to the road. Concerning the ownerless dogs, the community was the strongest variable, followed by human population density and proximity to temples. In contrast to owned dogs, residential area was a less important factor influencing the distribution of ownerless dogs.

**Table 3:** The variable importance obtained from the combined set of all study sites.

**Predictor variables**	**Owned dogs**			**Ownerless dogs**		
	**Count model**		**Binary model**	**Count model**		**Binary model**
	%IncMSE^*^	IncNodePurity^**^	**IncNodePurity**	**%IncMSE**	**IncNodePurity**	**IncNodePurity**
Aquaculture	1.792	1.305	6.940	−3.458	0.067	1.579
Community	3.360	1.329	3.420	10.375	1.907	4.043
Proximity to school	7.348	20.163	52.344	−1.053	5.851	32.046
Proximity to temple	6.516	22.242	50.707	5.479	7.531	29.308
Proximity to road	10.174	10.745	66.909	−3.799	2.469	16.997
Human population	36.581	47.463	149.117	9.933	10.867	68.335
Resident area	44.142	14.542	12.762	−2.258	0.892	5.012
Field crop	4.462	2.519	5.971	0.605	0.657	3.961
Horticulture	7.028	1.207	2.541	−1.188	0.419	2.043

* *%IncMSE is the increase in the mean square error of prediction, which the higher number, the more important variable is. It is the most robust and informative measure*.

** *IncNodePurity relates to the loss function which by best splits are chosen, which more useful variables achieve higher increases in node purities*.

The association between the fitted function and the predictor variables in owned dogs is shown in Supplementary Figure 1 (quantitative RF) and Supplementary Figure 2 (binary RF). These plots demonstrate that aquaculture, community, residential area, field crop, and horticulture had a positive association with the predicted values. Interestingly, human population density showed different associations at different densities. First, a negative association with the fitted function was found until reaching approximately 100 persons/km^2^. The association then returned to a positive trend, which rose to the highest point at 1,000 persons/km^2^. Finally, it turned back down again when the density exceeded that peak. Three proximity variables, including proximity to school, Buddhist temple, and road, showed a similar trend with two different association patterns, starting with a negative association to a certain level and turning back to a positive direction for a higher distance.

The association between the fitted function and the predictor variables for the ownerless dogs is shown in Supplementary Figure 3 (with quantitative RF) and Supplementary Figure 4 (with binary RF). Four variables, including community, resident area, field crop, and horticulture, showed a positive association. Aquaculture showed a negative association, while proximity to school, Buddhist temple, and road first showed a negative association and then turned back to be positive at a certain level. In contrast, the human population density initially showed a positive association and became negative when the density reached 1,000 persons/km^2^.

All the count and binary models showed a high predictive power for the training sets, whereas the evaluation of the final outputs by quantifying the GOF between the observed values of test sets and predictive values showed medium to low accuracy (Table 4). Regarding the count models of the training sets, the correlation varied in the range of 0.910–0.936, while the RMSE varied from 0.16 0.19. The binary models of the training sets also showed a high predictive power with an AUC ranging between 0.992 and 0.998, and the correlation ranged from 0.868 to 0.913. With regard to the evaluation of the final outputs, the accuracy of the predictive values in owned dogs was higher than that of ownerless dogs. The correlation ranged between 0.451 and 0.634 for owned dogs and from 0.101 to 0.471 for ownerless dogs. For the RMSE, the values ranged between 0.36 and 0.52 for the owned and from 0.16 to 0.38 for the ownerless dogs.

**Table 4:** Goodness of fit of the models.

Model^*^	**Response variables**	**Training sites**				**Test site**	
		**Count model**		**Binary model**		**Correlation**	RMSE^*^
		**Correlation**	RMSE^**^	AUC^***^	**Correlation**		
I	Owned dogs	0.933	0.17	0.992	0.887	0.516	0.51
	Ownerless dogs	0.910	0.16	0.992	0.888	0.319	0.38
II	Owned dogs	0.936	0.17	0.997	0.909	0.580	0.52
	Ownerless dogs	0.919	0.19	0.997	0.909	0.335	0.34
III	Owned dogs	0.936	0.17	0.993	0.884	0.634	0.36
	Ownerless dogs	0.914	0.18	0.993	0.885	0.101	0.16
IV	Owned dogs	0.934	0.17	0.998	0.913	0.451	0.51
	Ownerless dogs	0.921	0.17	0.997	0.911	0.472	0.30
V	Owned dogs	0.934	0.17	0.994	0.896	-	-
	Ownerless dogs	0.927	0.18	0.996	0.868	-	-

* *Details of each model are described in Table 1*.

** *Root mean square error (RMSE)*.

*** *Areas under the curve (AUC)*.

The total number of dogs in the entire country was calculated using our models. The lowest value was observed in Model I, whereas Model III showed the highest values (Table 5). On average, the total number of dogs was 12,840,452 (10,251,591–15,836,093 heads). Of these, 11,196,042 heads (ranging from 9,176,028 to 13,676,025) were owned with the density of 21.7 heads/km^2^ (ranging from 0 to 251.6 heads/km^2^). The estimated number of ownerless dogs was 1,644,419 heads (ranging from 1,075,563 to 2,256,904) with the density of 3.2 heads/km^2^ (ranging from 0 to 5.6 heads/km^2^).

**Table 5:** Predicted number of dogs in Thailand.

**Response variables**	**Predicted number of dogs**				
	**Model I**	**Model II**	**Model III**	**Model IV**	**Model V**
Owned dogs	9,176,028	12,137,153	13,676,025	11,266,308	11,196,042
Ownerless dogs	1,075,563	2,256,904	2,160,068	1,431,072	1,644,419
Total dogs	10,251,591	14,394,057	15,836,093	12,697,380	12,840,452

**Figure 4:**
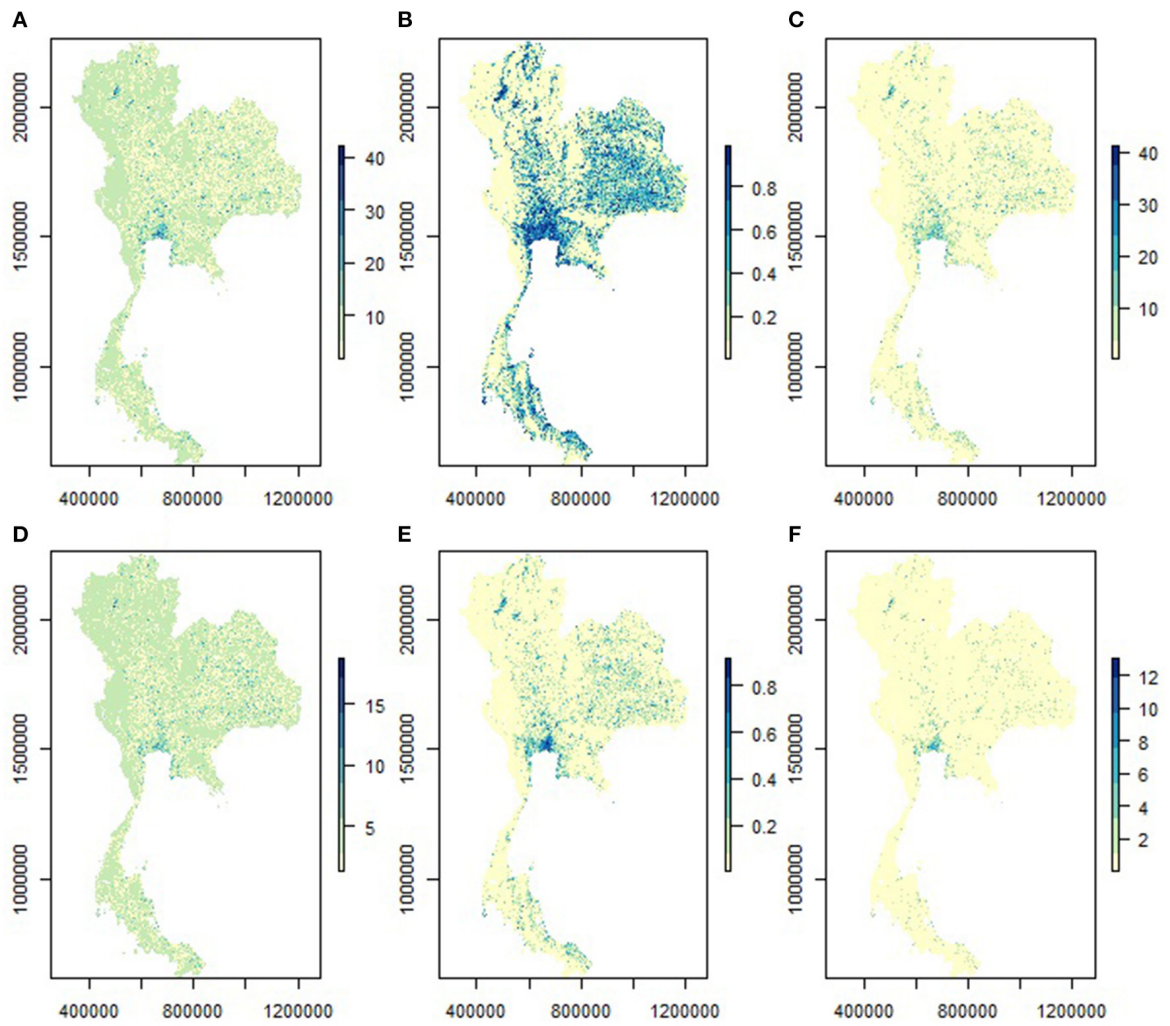
The predicted distribution of dog population in Thailand (300-m resolution). **(A)** Owned dogs with count model, **(B)** owned dogs with a binary model, **(C)** owned dogs with combining the two models **(A,B)**, **(D)** Ownerless dogs with count model, **(E)** Ownerless dogs with a binary model, **(F)** Ownerless dogs with combining the two models **(D,E)**.

**Figure 5:**
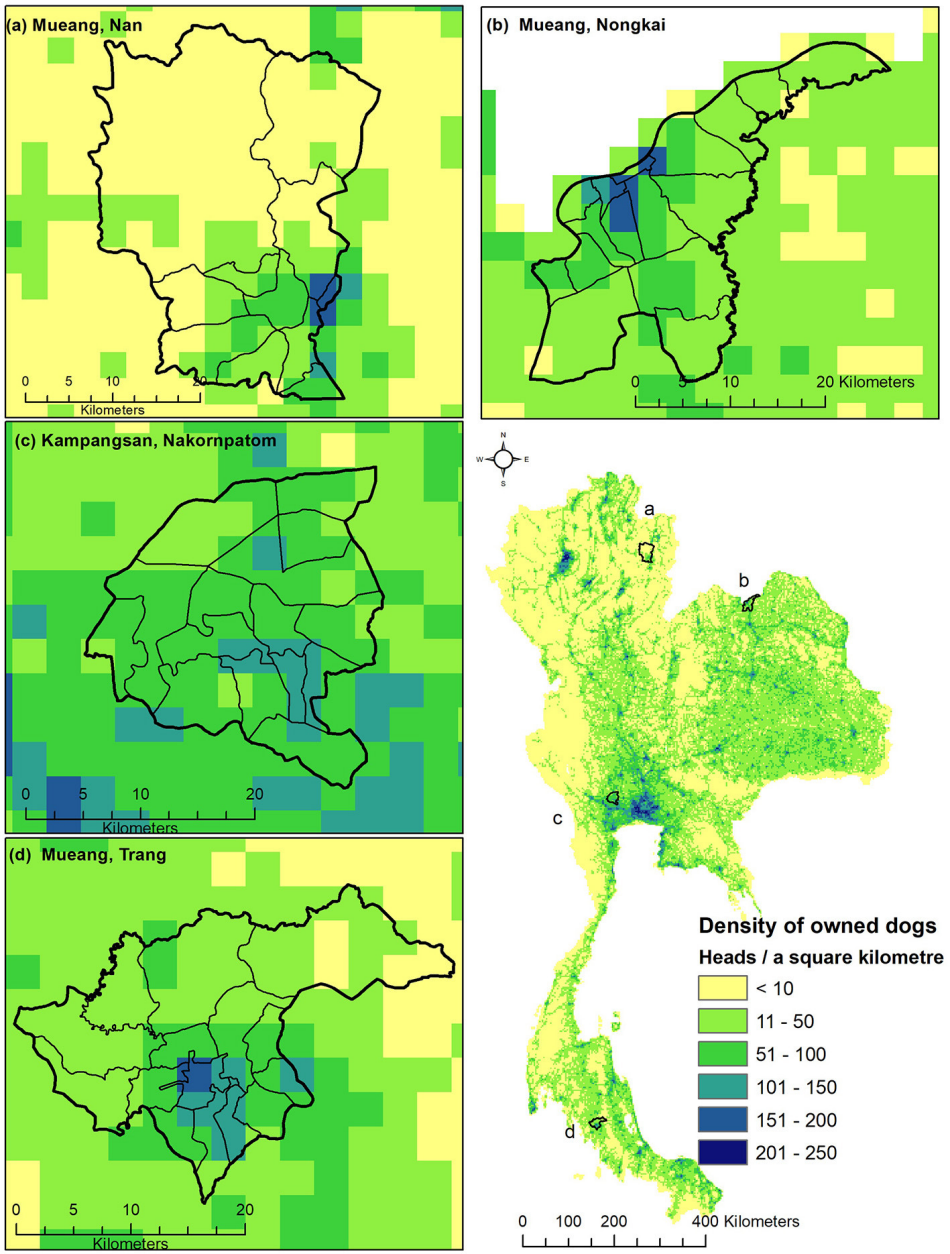
The distribution map of owned dog population in Thailand (1,000-m resolution). The distribution maps of the owned dog population in Muang Nan district, Nan province **(A)**, in Muang Nong Khai district, Nong Khai province **(B)**, in Kampangsan district, Nakhon Pathom province **(C)**, Muang Trang district, Trang province **(D)**, and the whole country (bottom right).

**Figure 6:**
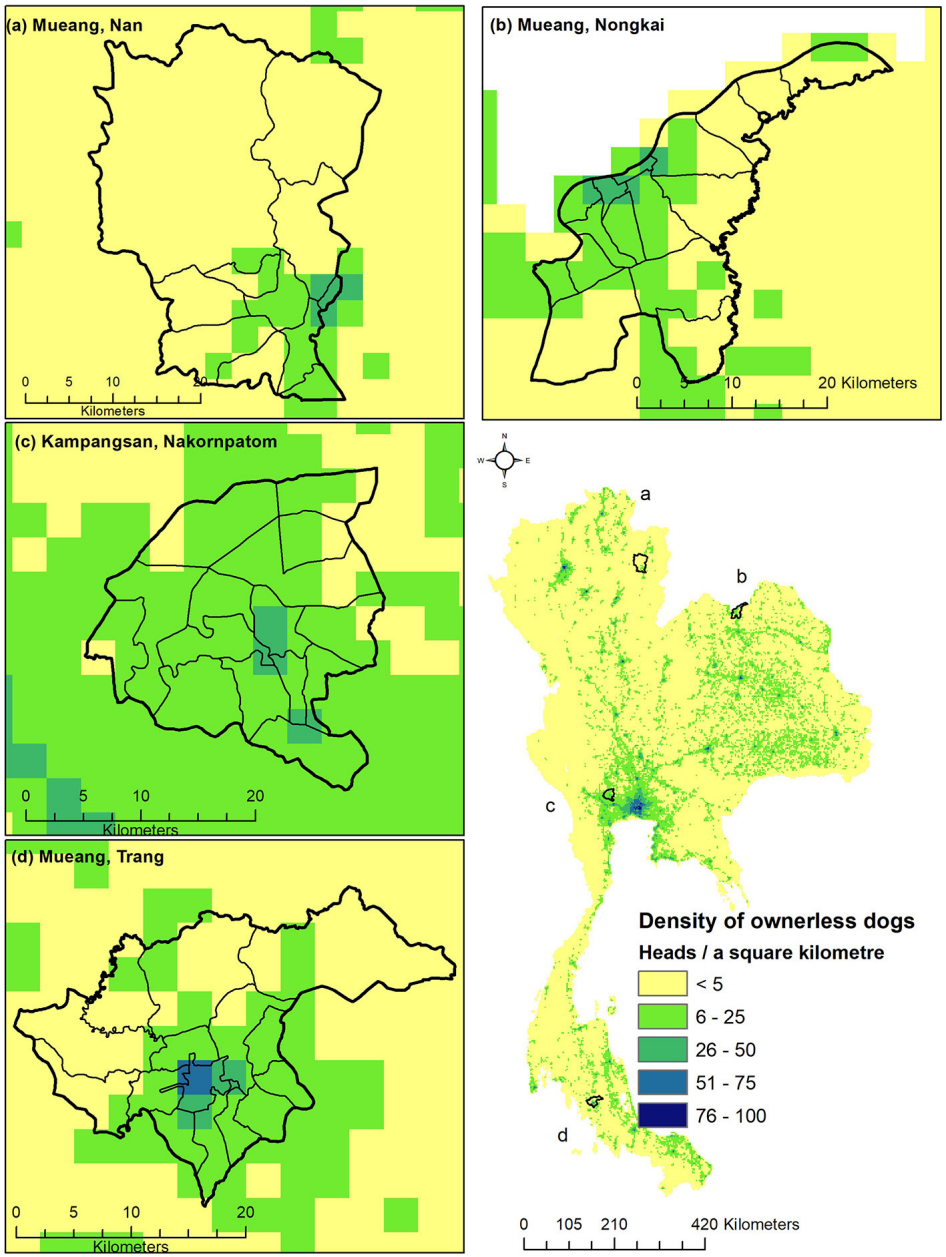
The distribution map of the ownerless dog population in Thailand (1,000-m resolution). The distribution maps of the ownerless dog population in Muang Nan district, Nan province **(A)**, in Muang Nong Khai district, Nong Khai province **(B)**, in Kampangsan district, Nakhon Pathom province **(C)**, Muang Trang district, Trang province **(D)**, and the whole country (bottom right).

The spatial distribution of owned and ownerless dog populations in Thailand, separated by the types of modeling approaches, are shown in Figure 4. The distribution maps of the owned and ownerless dog populations predicted by the combined models are shown in Figures 5, 6, respectively. It appears that the spatial distribution of owned and ownerless dogs had a similar pattern where the high-density areas were mostly distributed in the big cities, namely Bangkok (148 heads/km^2^ owned dogs and 42 heads/km^2^ ownerless dogs), Nonthaburi (136 heads/km^2^ of owned dogs and 32 heads/km^2^ of ownerless dogs), Samut Prakan (101 heads/km^2^ owned dogs and 22 heads/km^2^ ownerless dogs), and Pathumthani (95 heads/km^2^ of owned dogs and 16 heads/km^2^ of ownerless dogs). Focusing on the studied districts (Figures 5, 6), it was found that the urban areas showed a higher density of dogs compared to the rural areas.

## Discussion

This study integrated two techniques to estimate the number of dog populations in Thailand, including dog surveys and spatial modeling. Dog surveys throughout the country have been routinely conducted by local government organizations twice a year and reported to a web-based reporting system called “ThaiRabies.net” (27). However, such surveys face different challenges, such as technical problems within the system and incomplete data entry, resulting in unreliable data for dog populations. In this study, we selected the study areas at the district level, partitioned the selected districts into grids with a 300-m resolution, and surveyed the number of dogs in these chosen grids considering ownership statuses and movement restriction. A spatial modeling technique (40–42) was then used to predict the number of dogs using data from the surveys. This method also determined the factors influencing the spatial distribution of these dogs. Mark-recapture techniques were used in a previous study (29). Nonetheless, such methods are relatively labor-intensive and not extrapolatable to large areas (1). Therefore, what we developed here, which is applicable to the whole country, uses much less budget as well as manpower compared to manual dog surveys. This should be considered an alternative nationwide survey method.

Effective dog population management can mitigate the adverse impacts caused by dogs, particularly stray ones (1, 3, 8, 9, 21). This study found that the ratio of owned and ownerless dogs was 6.4:1. Apart from ownerless dogs, most owned dogs (~75%) also roamed freely. Concerning rabies, if these stray dogs are not vaccinated for any reason, the chance for these dogs to become infected and circulate the virus in the areas is higher (26, 43). Our findings are in line with a previous rabies investigation report from endemic areas in Thailand, in which most of the affected animals were unvaccinated-owned dogs (70%) with the characteristics of independent or semi-independent roaming, while the rest were ownerless dogs without vaccination (44). Therefore, the dog population control program should be emphasized to promote responsible dog ownership to reduce the number of strays, especially ownerless dogs. The size of dog populations and their distribution estimated from our study are helpful in strategic planning for dog population control programs, such as surgical sterilization. It was well-documented in a previous study that a proper dog population control can reduce the prevalence of rabies in dogs (45). In addition, our findings can also improve canine rabies vaccine allocation as per dog heads estimated in each area. In a previous study, herd immunity derived from vaccination coverage of 60% was proven effective in the prevention and control of rabies (46). However, an annual vaccination coverage of at least 70% of the dog population is recommended by the WHO to prevent ongoing transmission and eradicate rabies (47). To achieve the herd immunity threshold, the dog population size must be precisely estimated. This study provides an initial technical approach to do so. Future studies may modify our technique or include more data to improve our model.

Dog habitats are related to sources of food, water, and shelter, which are essential factors for dog living. Our study indicates that the habitats of owned dogs were linked to household demographics (resident areas, human population, and proximity to the road, respectively), which can be the sources of those factors. In ownerless dogs, however, the habitats were related to the community (premises in the community, human population, and Buddhist temple, respectively). A community is composed of markets, garbage dumps, restaurants, and other premises related to human activities. These scenarios have also been observed in other developing countries where the dog population is not well managed (13, 48–50). In Thailand, Buddhist temples are important sources of food for stray dogs and, in many cases, temples are also shelters for abandoned dogs (3). A study conducted in Bali, Indonesia also reported that dogs were mostly found around temples (13).

Based on our estimation, dog populations were highly distributed in large cities across the country, such as the Bangkok Metropolitan Region and surrounding provinces, Chiang Mai (Northern), Chonburi (Eastern), and Songkhla (Southern). Focusing on our studied districts, it was obvious that the urban areas showed a higher density of dogs compared to the rural areas. For example, in Chiang Mai province in northern Thailand, the estimated dog density of Mueang Chiang Mai district as the urban area was 184 heads/km^2^ (143 heads/km^2^ of owned dogs and 41 heads/km^2^ of ownerless dogs), while the estimated dog density of Fang district in rural areas was 29 heads/km^2^ (25 heads/km^2^ of owned dogs and 4 heads/km^2^ of ownerless dogs). This was associated with household demographics, which has been reported in other studies (51, 52). The population distribution map we produced can be further used to predict the number of dogs by involving other factors such as population structures and dynamics, predict the occurrence of diseases such as rabies in dog populations, and use as baseline data for dog population management plans.

In this study, we used the random forest model with a two-part approach because of its high performance and the ability to handle zero inflation of the dataset. The RF model is a machine learning method with a non-parametric approach. The algorithms combine the prediction of a high number of classification trees in an ensemble (53). Compared to other methods, RF has a high capability to model complex interactions among predictor variables (54) and has recently provided highly accurate results in modeling livestock (41, 55) and human populations (56, 57). A two-part approach (36, 37, 58) was used to deal with many of the surveyed grids with dog absence (zero inflation), where the presence/absence was modeled separately from abundance (presence only). The absence of dogs in the census grids may have occurred due to unsatisfactory conditions for dog living (structural unsuitability); satisfactory conditions but dogs were temporarily absent at the time of survey (design error); satisfactory conditions and dogs were present, but the observers misidentified or missed their presence (observer error); and satisfactory conditions but with no attendance (human error). The zeros due to design, observer, and human errors are also called false zeros or false negatives, and the structural unsuitability is called positive zeros, true zeros, or true negatives (59). The limitation of the two-part models applied in this study is that the four different types of zeros are not distinguishable (59). However, based on a previous comparative study on five regression models (Poisson, negative binomial, quasi-Poisson, two-part model, and zero-inflated Poisson), it was found that the two-part model is still the most promising method (36).

This study has some potential limitations. First, we may not have reached an appropriate sample size. The 400 sample grids with 300-m resolution located in four districts across the country might be too small compared to the entire territory. Nonetheless, the manual survey at the national level is not practical in the long run, as it is labor-intensive, costly, and time-consuming. Our spatial modeling is an alternative to solve this problem, even though the results may not perfectly reflect the reality. A greater number of sample grids is required in future studies to improve the model performance. However, it is still not practical to conduct manual surveys. Pattern recognition or biometric technology is suggested for automatically identifying and distinguishing individual dogs in the survey. With such a technological approach, the number of survey grids would be dramatically increased with less effort, and a more accurate estimation would be generated.

We estimated approximately 12.8 million dogs across Thailand. This estimation seems plausible. A previous study conducted in 1994 reported approximately 7.3 million dogs nationwide (60), and given that the population growth rate is 1–2% per year (61, 62), there should be approximately 9.5–12.5 million heads in 2020. However, direct model validation and verification are not possible because of the lack of actual field data. A nationwide survey using the aforementioned technology is needed at least once to assess model accuracy.

Dog population management is a keystone in addressing dog-related problems, especially in stray dogs. Responsible dog ownership should be rigorously promoted as well as the control of stray dog populations to an acceptable level to improve their health and welfare simultaneously. The community should be involved in promoting responsible dog ownership, which can help reduce the number of ownerless dogs. Better veterinary care should be employed by improving the health of individual animals and increasing dog vaccination coverage, which could reduce rabies transmission (45). With these approaches, the elimination of rabies in dog populations is possible. Moreover, knowledge of the population size and spatial distribution of dogs can facilitate the implementation of mass dog vaccination campaigns and stray dog population control programs to control canine rabies, which will greatly contribute to the elimination of rabies in Thailand by 2030.

## Data Availability Statement

The data that support the findings of this study are available from the Department of Livestock Development by restrictions apply to the availability of these data, which were used under license for the current study, and so are not publicly available. Data are however available from the authors upon reasonable request and with permission of the Department of Livestock Development. Requests to access the datasets should be directed to weeraden@yahoo.com.

## Ethics Statement

Ethical review and written informed consent for participation were not required for the animal study because this research is on spatial modeling and population estimation of dogs, that cannot link to individual animal subjects.

## Author Contributions

WT, SK, and KL conceived and designed the study. SK, VW, KB, TP, KL, and WT conducted dog surveys and generated the raw data. WT performed statistical analysis with contributions from MG. WT drafted the paper which was meticulously revised by AW and KL. WT, KL, and AW contributed to writing, reviewed, and editing the final manuscript. All authors read and approved the published version of the manuscript.

## Funding

This work was financially supported by the project Saving Animals and Human Lives from Rabies Project following the Determination of Professor Dr. Her Royal Highness Princess Chulabhorn Mahidol.

## Conflict of Interest

The authors declare that the research was conducted in the absence of any commercial or financial relationships that could be construed as a potential conflict of interest.

## Publisher's Note

All claims expressed in this article are solely those of the authors and do not necessarily represent those of their affiliated organizations, or those of the publisher, the editors and the reviewers. Any product that may be evaluated in this article, or claim that may be made by its manufacturer, is not guaranteed or endorsed by the publisher.
