# Supplementary material for: Spatial Distribution and Population Estimation of Dogs in Thailand: Implications for Rabies Prevention and Control
Source: Front Vet Sci. 2021 Dec 21;8:790701. doi: 10.3389/fvets.2021.790701 (PMC8724437; doi:10.3389/fvets.2021.790701)
Supplement: Supplementary file 1 [file Data_Sheet_1.PDF]

Supplementary Figures

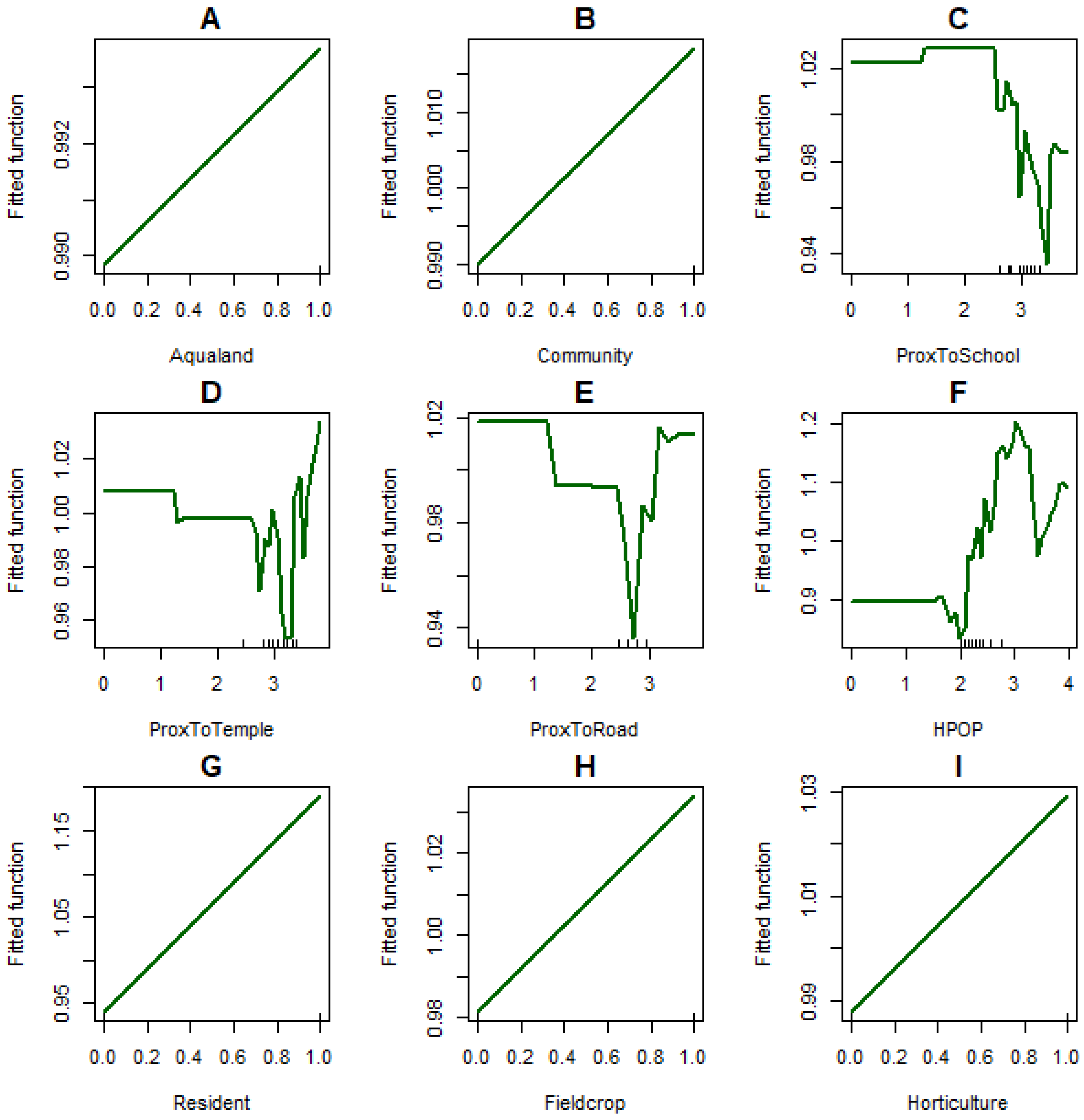

Supplementary Figure 1. Association between the fitted function and predictors of quantitative RF for owned dogs

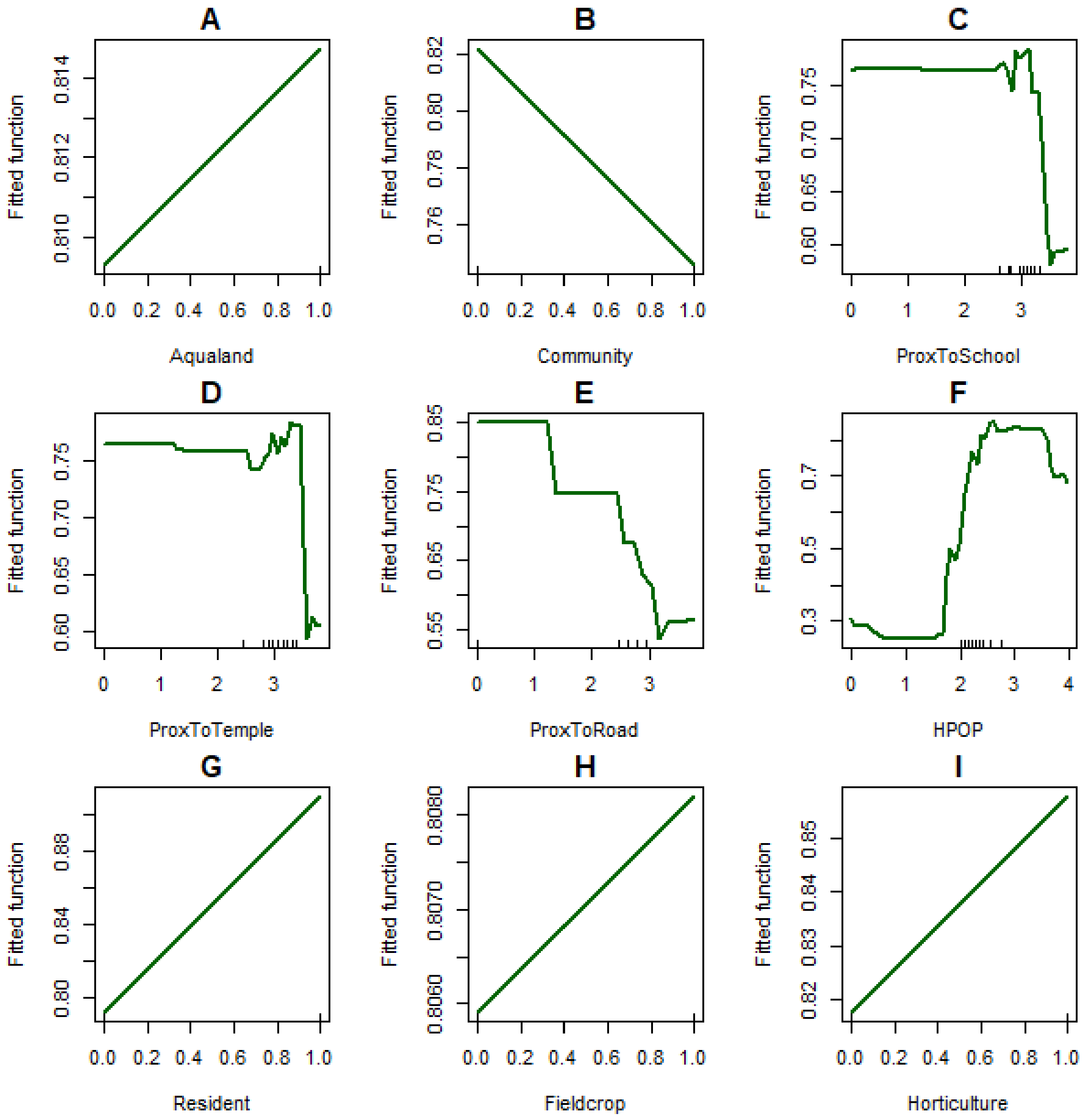

Supplementary Figure 2. Association between the fitted function and predictors of binary RF for owned dogs.

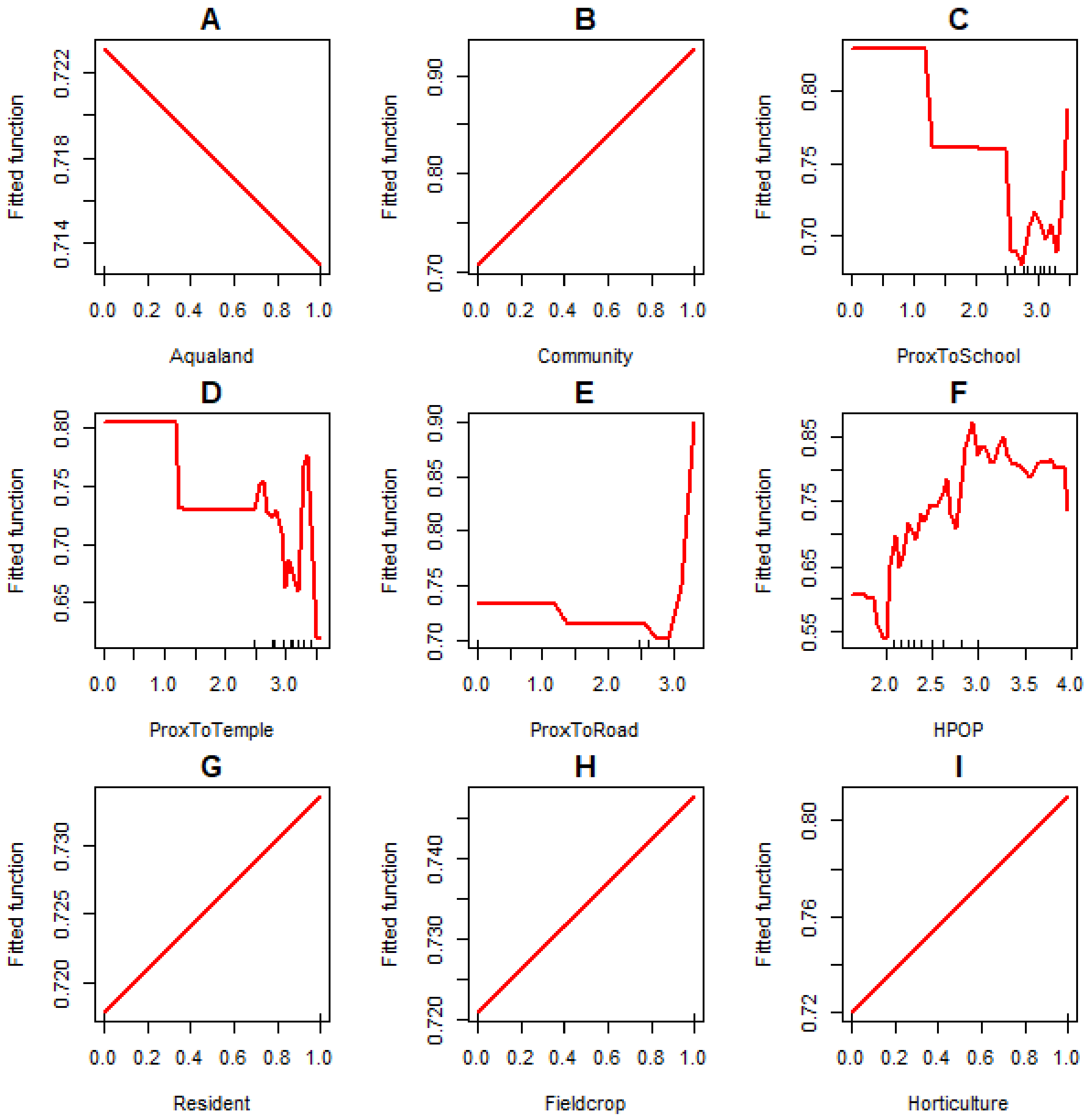

Supplementary Figure 3. Association between the fitted function and predictors of quantitative RF for ownerless dogs

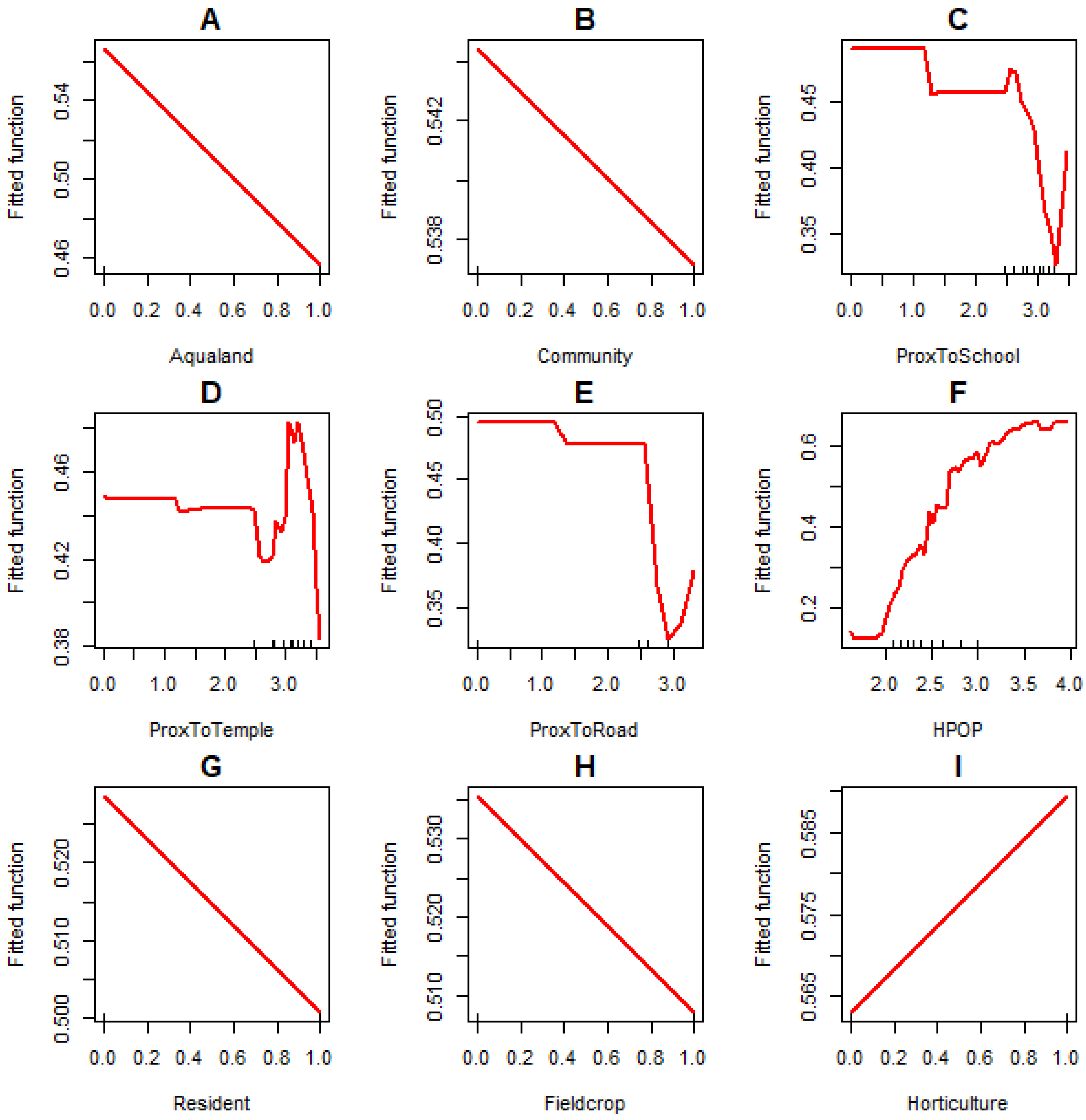

Supplementary Figure 4. Association between the fitted function and predictors of binary RF for ownerless dogs
